# Co-creating holistic injury prevention training for youth handball: Development of an intervention targeting end-users at the individual, team, and organizational levels

**DOI:** 10.1186/s13102-023-00800-6

**Published:** 2024-01-08

**Authors:** Eva Ageberg, Sofia Bunke, Jennie Linnéll, Karin Moesch

**Affiliations:** 1 Department of Health Sciences, Faculty of Medicine, Lund University, PO Box 117, Lund, SE-221 00 Sweden; 2 Department of Psychology, Lund University, Lund, Sweden; 3 Swedish Handball Federation, Stockholm, Sweden; 4 Department of Sports Sciences, Malmö University, Malmö, Sweden

**Keywords:** Adolescent, Team sports, Preventive therapy, Sports medicine, Sport psychology, Resistance training

## Abstract

**Background:**

Interventions that are co-created with end-users, and that are informed by behavior change or implementation theories, support implementation in real world settings. However, injury prevention programs for youth athletes have typically been developed by experts with no, or insufficiently described, end-user involvement and without guidance by theories. The aim of the current study was to describe the development of a holistic injury prevention intervention for youth handball targeting end-users at different levels, through using knowledge from end-users and researchers/experts and applying relevant behavior change and implementation theories.

**Methods:**

Knowledge from researchers/experts (sports medicine, sport psychology, handball, physical therapy, strength and conditioning (n = 14)) and end-users (coaches, players, club administrators, n = 16), and applying relevant implementation (Consolidated Framework for Implementation Research, CFIR), behavior change (Health Action Process Approach, HAPA) and motivational (Self-Determination Theory, SDT) theories, were used to co-design the intervention. Early end-users (coaches (n = 6), players (n = 3) and a club administrator (n = 1)) were interviewed for initial feedback.

**Results:**

The intervention consisted of end-user-targeted information and training that was made available in a specifically developed interactive mobile application with modules for players, coaches, club administrators, and caregivers. Information for all end-users included benefits and principles of physical and psychological injury prevention training, load-management, motivation, and communication. Information about implementation was developed for club administrators specifically. For coaches, training to do with their teams included handball-specific injury prevention exercises (legs, shoulders, core) combined with psychological aspects (motivation, task focus, body awareness) to integrate within warm-up and handball skills training. Training for players included handball-specific multi-joint strength, power, and explosive exercises (legs, shoulders, core) and sport psychology exercises (self-awareness, relaxation, being in the present moment, prevent and handle stress). To support self-management, adoption, and motivation, programs were provided, and players and coaches could change, add, progress the difficulty of exercises, and build their own programs. Initial feedback from early end-users was generally positive.

**Conclusions:**

Utilizing an approach where researchers/experts and end-users co-created injury prevention training specifically for youth handball, an intervention was generated that included information and holistic training targeting end-users at the individual (players, caregivers), team (coaches), and organizational (club administrators) levels.

**Supplementary Information:**

The online version contains supplementary material available at 10.1186/s13102-023-00800-6.

## Background

Research has established that physical and psychological injury prevention training is effective in reducing injuries in youth team sports [1, 2], but public health impact tends to be limited because such training is not widely or properly implemented over a sustained period of time. Insufficient involvement of end-users at the individual and organizational levels has been identified as a main barrier to implementation [3], while interventions that are co-created with end-users support implementation in real world settings [4].

In previous studies, injury prevention programs for youth athletes have typically been developed by experts with no, or insufficiently described end-user involvement [5–7]. It is suggested that end-users are involved in the development of programs to ensure they are sufficiently context- and content-specific [3, 8, 9]. In more recent studies, injury prevention training has been co-created with end-users for youth [10] and adult women [11] athletes, although it remains to be evaluated whether these programs will enhance adoption and sustained use [10, 12]. While coaches are key to deliver injury prevention training, behavior change is required among numerous stakeholders, including players, coaches, and organizational representatives, to integrate injury prevention training into regular training routines [13, 14]. It is also emphasized that behavior change or implementation theories inform the intervention to facilitate implementation [15].

The ‘Implementing injury Prevention training ROutines in TEams and Clubs in youth Team handball (I-PROTECT)’ project was initiated through dialogue between end-users and researchers with the goal of making injury prevention training an integral part of regular practice in youth handball through a series of studies [16]. Based on identified facilitators to support the implementation of injury prevention training [17], researchers/handball experts and end-users co-created a first pilot version of handball-specific injury prevention exercises for coaches to use with their team(s) in warm-up and skills training [10]. It was identified that end-users wanted example programs, clear instructions (e.g., why and how to perform exercises, movement technique, feedback) and requested the intervention in a digital platform [10]. Coaches and players believed the pilot program was relevant, meaningful, and useful but wanted more variations of exercises and programs with fewer exercises [18]. Also, further work was needed to develop handball-specific injury prevention strength exercises and sport psychology exercises [10], as well as information targeting all stakeholders [10, 17], including caregivers [19]. Therefore, the aim of the current study was to describe the development of a full version of the I-PROTECT intervention targeting all end-users, i.e., players, coaches, club administrators, and caregivers, through using knowledge from end-users and researchers/experts and applying relevant behavior change and implementation theories.

## Materials and methods

This study adheres to the Standards for Reporting Qualitative Research (SRQR) (https://www.equator-network.org/reporting-guidelines/srqr/). Preliminary data were presented as abstracts at scientific conferences 2023 [20, 21].

### Guiding theories

Identified barriers and facilitators from our previous studies within I-PROTECT [10, 17, 18] informed the development of the content (information, physical and sport psychology exercises) and delivery of the intervention, categorized according to relevant topics in the Innovation domain of the updated Consolidated Framework for Implementation Research (CFIR) [22]. Barriers were mainly related to lack of knowledge and time, while facilitators were principally about to be well informed, have end-user-targeted information and training, and have programs with selected exercises along with the option to choose exercise/level of progression (Appendix A).

The behavioral theory Health Action Process Approach (HAPA) [23], and the motivational theory Self-Determination Theory (SDT) [24], guided the development of information and exercises to facilitate behavior change and motivation. The HAPA theory [23] was used to ensure motivational and volitional (action) strategies. SDT was used to promote high quality motivation by considering the three basic needs autonomy, perceived competence, and relatedness [24]. In addition, goal setting principles [25] were applied to develop information about implementation for club administrators.

### Information

The research team developed information regarding importance, benefits, and principles of injury prevention training, load management, and communication and feedback targeting the different end-users. Also, information about implementation was developed for club administrators. The research team (authors of this paper) represented different expert fields (both theory and practice) as follows: EA was a senior researcher with a PhD in medical sciences and expertise in physical therapy and sports medicine, and 12 years of clinical experience in sports injury rehabilitation. KM was a junior researcher with a PhD in sport sciences and a PhD in sport psychology, and 19 years of clinical experience as a sport psychology consultant. SB was a junior researcher with a PhD in sport psychology and 12 years of experience working with youth as a teacher in a sports high school. JL had an MSc in sport psychology, experience as an elite handball player (13 years), coach (8 years), coach educator (8 years) and sport psychology consultant (2 years).

### Training

**Fig. 1:**
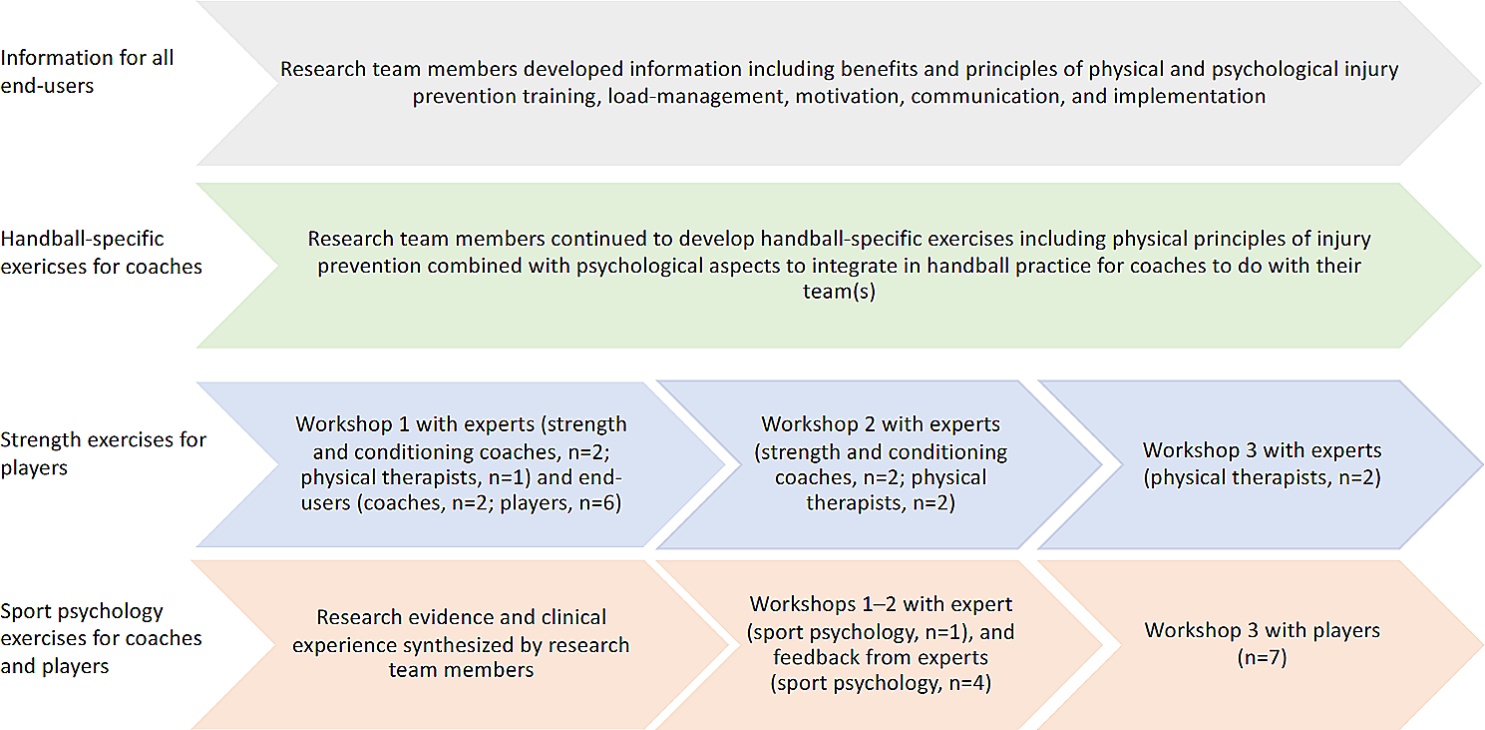
Development of the intervention consisting of end-user targeted information and training, based on previous studies within I-PROTECT [10, 17, 18], and guided by the behavior change (Health Action Process Approach, HAPA) [23] and motivational (Self-Determination Theory, SDT) theories [24]

Handball-specific exercises including injury prevention principles, injury prevention strength exercises (body weight for young players, weight training for older players), and sport psychology exercises were developed, based on the needs identified in previous studies [10, 18]. Figure 1 provides an overview of the development of the intervention (information and training).

### Handball-specific exercises

Building on previous results [10, 18], research team members (JL, EA) continued to develop handball-specific exercises including physical principles of injury prevention (movement technique upper and lower extremities, respectively, and muscle strength) combined with psychological aspects (increase end-user motivation, task focus and body awareness) to integrate in handball practice warm-up and skills training for coaches to do with their team(s).

### Strength exercises

Three workshops were held to develop handball-specific strength exercises. Three authors (EA, JL, SB) prepared the workshops and invited players, handball coaches, strength and conditioning coaches, and physical therapists to participate. Given our previous experience with conducting workshops [10], and that players and coaches are generally well familiar with strength training, the research team decided to have a first workshop with experts and end-users together. The subsequent workshop built on the previous one.

In the first workshop (face-to-face, February 2020), participants developed and tested injury prevention principles and exercises that youth handball players (15–17 years) could integrate within their strength training. All participants had experience in handball. Participants who volunteered to participate were: Six youth players (3 male and 3 female, all aged 16–17 years) and two coaches (1 man and 1 woman; one was a clinically active physical therapist and a strength and conditioning coach in a handball club, and the other was a physical therapy student) recruited from teams with players 15–17 years of the two clubs participating in the I-PROTECT study [16]; one clinically active physical therapist and former elite handball player (female); and two strength and conditioning coaches (2 men, both worked at a gym and had extensive experience in strength and conditioning training for youth and elite handball players). Three authors (EA, JL, SB) led and facilitated the 2.5-hour structured workshop.

The workshop started with an introductory didactic session to provide participants with information about the I-PROTECT project, and results from previous studies within the project, including the mobile application I-PROTECT GO [10, 17, 18]. Participants were then divided into four groups: (1) Female players (n = 3), (2) Male players (n = 3), (3) Handball coach, strength and conditioning coach, and physical therapist (n = 3), and (4) Handball and strength and conditioning coaches, and one author who was also a handball coach and former elite player (n = 3). The other two authors circulated between groups 1, 2 and 3, to answer questions and/or reinforce the task(s) and goal of the workshop. The players (groups 1 and 2) were asked to discuss what type of strength exercise content players want/need, how they wanted this material to be presented, how they believed that strength training could be presented/delivered to enhance motivation for uptake, and to provide examples of exercises. Group 3 was asked to focus on the lower extremities and group 4 on the upper extremities and core. Groups 3 and 4 were asked to provide examples of exercises to improve strength and reduce the risk of injury, emphasize important injury prevention principles, and describe important general exercises. Workshop participants documented their results, presented results in the whole group, and gave each other input. Research team members documented results from the discussions in small groups and the whole group. Based on the results from this workshop, research team members (JL, EA) continued to develop strength exercises and planned the following workshop.

In the second workshop (held over Zoom, February 2021), strength programs were developed. A 3-hour workshop was led by two authors (JL, EA). Two strength and conditioning coaches (men) and two clinically active physical therapists specializing in sports medicine (1 woman, 1 man) participated. All four had handball knowledge. Participants were provided with a summary of results from previous I-PROTECT studies, results from the first workshop, and questions to discuss, before the workshop. The two strength and conditioning coaches participated the first 1.5 h to discuss setup and structure of programs, exercises, instructions, programming (sets, repetitions, time, load), and periodization. The two physical therapists joined the last 1.5 h of the workshop, in which participants were divided into two groups to discuss the bank of exercises with focus on the content (strength and conditioning coaches) and principles of injury prevention (physical therapists), respectively. Each group then summarized their discussions in the whole group.

More time was required to discuss all exercises from principles of injury prevention. Therefore, a third 1-hour workshop (face-to-face, April 2021) was held with the two physical therapists by one author (JL) in which the remaining exercises were discussed. Also, strength exercises (body weight or low weights/resistance) for players 13–14 years were discussed.

The authors documented and synthesized the results. The experts were asked to provide any feedback on the synthesis. A film production company was appointed to produce videos of exercises, and elite/professional and youth players were recruited to demonstrate the exercises.

### Sport psychology exercises

Given that players and coaches were generally unfamiliar with sport psychology exercises, experts initiated the development of these exercises and end-users then tested and evaluated them. The three first steps of the generalizable six-step intervention development process [26] were followed: (1) identify and synthesize research evidence and clinical experience; (2) consult relevant experts and (3) engage end-users to ensure their needs, capacity and values were considered.

First, peer-reviewed literature was searched to identify potential sport psychology exercises. In this literature search, central publications on the topic in focus (i.e., injury prevention and psychological interventions for injury prevention) were identified. Also, potential interventions within the topic stress reduction and within the focus areas self-reflection and self-awareness, interpersonal knowledge, arousal regulation, mindfulness training, psychoeducation (e.g., the importance of recovery), motivation and goalsetting, all of them emerging from the results of the sport psychology expert workshop 2018 [10], were identified. The synthesis of the results was guided by one author’s (KM) clinical experience and consisted of a first version of principles and exercises for injury prevention from a psychological perspective. Regular meetings were held, in which two authors (SB, JL) provided feedback on the process, and decisions were discussed.

Second, experts in sport psychology were consulted to evaluate the exercises that were developed in step 1 to ensure quality of the content. Players’ exercises and exercises for coaches to do with their team(s) were presented and discussed in two separate workshops (a 2-hour online workshop about players’ exercises, December 2020; a 1.5-hour online workshop about coaches’ exercises, January 2021) with one of the experts who participated in a previous I-PROTECT study [10]. Discussions centered around the meaningfulness and feasibility of the exercises, as well as the age appropriateness of the exercises. This expert was chosen as he had expertise in both sport psychology (PhD, and sport psychology consultant) and handball (youth coach). Both workshops were led by two authors (KM, SB). The sport psychology experts (n = 4) who participated in the previous I-PROTECT study [10], and were not part of the research team, were asked to provide feedback on the sport psychology principles and exercises for injury prevention.

Third, a workshop with handball players from an elite sport high school was conducted to receive feedback about the sport psychology exercises for players. All students in the second and third years (n = 18) were invited and seven female players, all aged 18 years, agreed to participate. A 2-hour workshop was conducted online (December 2020), led by two authors (KM, JL). After a short introduction with information about the I-PROTECT project, four exercises were presented to the participants. The participants were then asked to discuss these exercises and provide feedback about (i) content; (ii) instructions; (iii) potential improvements, and (iv) appropriate age span for each exercise.

### Mobile application

As requested by end-users [10, 18], we continued to develop the mobile application (I-PROTECT GO), building on the first prototype [10]. While the first prototype targeted coaches only [10], modules for players, coaches, club administrators, and caregivers was developed in the present study. Information Technology consultants were appointed to produce the mobile application.

### Feedback from early end-users

Six coaches, three players and one club administrator, previously not involved in the I-PROTECT project, tested the intervention for 6 weeks. They were interviewed (data from pilot study, ClinicalTrials.gov Identifier: NCT05304507) about their experiences about the content, delivery, and implementation of the intervention. Participants were also asked to provide any ideas for future improvement to help implementation of the intervention.

The six coaches (two female and four male coaches representing four different teams) participated in one of two focus group interviews shortly after the intervention was completed (May 2022). The interviews were led by two authors (JL on site and KM online). The three players (males aged 14 years from the same one team) participated in individual interviews held online (KM) after their team had tested I-PROTECT GO (March 2023). The club administrator was interviewed (May 2022) online (EA, JL).

The procedure and analysis were similar for the focus group and the individual interviews. Participants were first informed about the topic and the process of interviews. Based on an interview guide, the participants were then asked to share their experiences of the content of the intervention, and their general opinion of the mobile application. Follow-up questions were used to encourage participants to expand on responses and to further gather participants’ experiences of facilitators and barriers for sustained use. The focus group interviews with coaches lasted 55 and 57 min, respectively, the three individual interviews with players lasted between 30 and 35 min, and the interview with the club administrator lasted 51 min. All interviews were video and audio recorded. The interviews with coaches and players were transcribed verbatim, and participants were deidentified in the interview transcripts. Data analysis was guided by the principles of the general inductive approach [27] by one author (KM) using QSR International’s NVivo 12 qualitative data analysis software (released March 2020), and another author (SB) acted as critical friend for the analysis. The interview with the club administrator was summarized descriptively (EA, JL) regarding barriers and facilitators for content, delivery, and implementation of the intervention, respectively.

## Results

### Information

Basic information about the I-PROTECT project and current knowledge about benefits and principles of physical and psychological injury prevention training was developed for all end-users. The motivational strategies of HAPA were applied, e.g., as follows: information that injuries are common (risk perception), that injury prevention training can reduce injuries by approx. 50% (outcome expectancies), and that injury prevention skills can be improved by practice (action self-efficacy). Additional information was developed to target each end-user group, e.g., load-management, motivation, and communication. Information about implementation for club administrators included key components of strategies, processes, and actions for implementation, e.g., describe goals, identify possible barriers and solutions to address these, describe follow-up, maintenance, activities, roles and responsibilities, aligned with the HAPA volitional strategies (action- and coping planning, maintenance- and recovery self-efficacy).

### Training

In general, the volitional strategies of HAPA were applied for the exercises as follows: clear instructions on when, where, and how (action planning) and handball-specific exercises with variations and progression to support sustained use (maintenance- and recovery self-efficacy). Also, the three basic needs of SDT were reinforced in exercises as follows: option to choose exercise and to encourage own initiatives (autonomy), clear purpose of exercise and focus on constructive task-oriented feedback (competence), and reinforce positive team behaviors, role models, pairwise/group exercises, and/or competition (relatedness).

### Handball-specific exercises

Handball-specific injury prevention exercises were developed to be included in warm-up (n = 23) and handball skills training (n = 34). The exercises targeted the legs (n = 30), shoulders (n = 16), and core (n = 11). Forty-three exercises targeted both younger (13–14 years) and older (15–17 years) players and 14 exercises only older players. The final version of the handball-specific exercises, along with a description of example programs, are provided in Table 1.

**Table 1:** Final version of handball-specific exercises (n = 57) to be integrated within warm-up or skills training. Exercises include physical principles (i.e., hip-knee-foot alignment, soft and controlled landings, muscle strength legs, core, posterior part of shoulder, wrist, grip) with integrated psychological aspects (i.e., pairwise, peer-feedback, competition, mindful muscle activation) agreed on among experts [10]. A handball is included in most exercises. A description of the example programs is displayed below the Table

Main body part (exercises, n)	Example of exercises	Level of progression (n)
Exercises integrated within warm-up (n = 23)		
Lower extremities (n = 11)	Run with foot plant; Lunges; Double-leg squats with partner in plank position; single-leg balance with drop and catch ball	Level 1 (n = 5), level 2 (n = 4), level 3 (n = 2)
Upper extremities (n = 6)	Shoulder external rotation exercises; Drop & catch ball; Shoulder press exercises	Level 1 (n = 6)
Core (n = 6)	Plank with arm wrestling and ball; Single-leg balance with ball pairwise; Slow-motion rotations–man-to-man	Level 1 (n = 4), level 2 (n = 1), level 3 (n = 1)
**Exercises integrated within handball skills training (n = 34)**		
Lower extremities (n = 19)	Jumping and cutting exercises with catch and throw	Level 1 (n = 9), level 2 (n = 6), level 3 (n = 3), level 4 (n = 1)
Upper extremities (n = 10)	Backwards throw; Throw behind back; Overhead-throw	Level 1 (n = 5), level 2 (n = 5)
Core (n = 5)	Core side to side with catch and throw; Core sit-up with catch and throw	Level 1 (n = 2), level 2 (n = 2), level 3 (n = 1)
Integrated strength (n = 6)	Strength exercises coupled with specific handball skills practice, i.e., shoulder– throwing; core– defence; jumping and landing– feint and cutting movements	NA

Example programs for the exercises were created and provided as follows:

• A set of 3 example programs (one program for each training session per week) provided every 6 weeks over 6 periods over the season, yielding a total of 18 example programs. That is, 3 programs delivered over 6 weeks, and then a new set of 3 programs delivered over 6 weeks, and so on

• Each program includes 5–7 physical exercises for legs, shoulders, and core, with:

o 4 exercises (2 legs, 1 core, 1 shoulder exercises) integrated in the warm-up

o 1–3 exercises integrated in the skills training as follows:

♣ Program 1: leg exercises

♣ Program 2: integrated strength. Specifically, a technique exercise and an injury prevention exercise (for legs, shoulder, or core) performed alternately to: increase players’ awareness of relation between performance (e.g., jump shot) and injury prevention (e.g., landing technique); reduce repetitive movements (e.g., shooting); use time at practice efficiently (i.e., avoid waiting in line); use the entire court (injury prevention exercises performed at the center, technique exercises by the 9- and 7 m lines); increase motivation in that players perform exercises in pairs (relatedness), choose when they do technique or injury prevention exercise (autonomy), and provide peer-feedback (competence)

♣ Program 3: shoulder exercises

• 1–2 alternative exercises (increased level of progression or different exercise for same body part, i.e., legs, shoulders, or core) provided for each exercise for variation

### Strength exercises

The results of the workshops are provided in Appendix B. In workshop 1, participants agreed on general principles for the lower and upper extremities, setup, type of exercises, and example of exercises for lower extremities, upper extremities, and core that were deemed relevant for youth handball players. Feedback from players regarding exercises and delivery to enhance motivation, that had not emerged in previous studies, included, e.g., exercises with free weights and that target muscle groups. In workshop 2, participants agreed on programming (sets, repetitions, time, load), number of programs, and number of exercises and alternative exercises in each program. In workshop 3, participants agreed on some additional principles and type of exercises, e.g., adding exercises in the frontal plane and for hip abductors. Strength exercises with body weight or low weights/resistance were developed for younger players. The final version of strength exercises (n = 82) for lower extremities, upper extremities, and core is provided in Table 2.

**Table 2:** Final version of handball-specific strength exercises (n = 82) for lower extremities, upper extremities, and core, along with examples of exercises and level of progression

Main body part (exercises, n)	Type/target of exercise	Example of exercises	Level of progression (n)
Lower extremities (n = 33)	Double-leg, single-leg, back thigh, front/inner thigh, hip abductors	Squats with kettlebells, dumbbells, barbell, trap-bar; step-ups, step-downs, lunges, split-squats; Nordic hamstrings, leg curl with gym ball, hip-thrust, Romanian deadlift, kettlebell swing, side lying hip abduction, Copenhagen adductor, slide exercises	Level 1 (n = 17), level 2 (n = 12), level 3 (n = 4)
Lower and upper extremities (n = 15)	Explosive strength	Double-leg and single-leg jump-landings from step-up board, box-jumps, skate-jumps, squat jumps, jump-landing on bosu-ball, long jumps, death jumps, dumbbell jerk, hang clean, sled push	Level 1 (n = 8), level 2 (n = 5), level 3 (n = 2)
Upper extremities (n = 25)	Front and back	External rotation, push press (lying, sitting, standing) with different equipment; pull exercises (sitting, standing) with different equipment; chins	Level 1 (n = 12), level 2 (n = 8), level 3 (n = 5)
Core (n = 9)	Strength and stability	Core wheel with gym ball or barbell, rotational core exercises (sitting standing), dead bug	Level 1 (n = 6), level 2 (n = 2), level 3 (n = 1)

### Sport psychology exercises

Nine intervention studies, six overviews (critical reviews, systematic reviews and meta-analyses), and a workbook focusing on stress reduction for adolescents were identified (Appendix C). This literature together with one author’s (KM) clinical experience, resulted in a list of 60 sport psychology exercises that centred around the following principles: self-awareness, being in the present moment, relaxation, and prevent and handle stress. From these 60 exercises, a selection of exercises was made based on: (i) most central for the intervention; (ii) easiness to present in a mobile application; and (iii) relevance to players’ age. A total of 11 exercises for coaches to do with their team(s), and 26 exercises for players, were selected (Table 3).

**Table 3:** Principle, aim, rationale, and examples of sport psychology exercises for coaches and players developed by research team members and evaluated by experts and end-users (players)

Principle	Aim	Rationale	Example exercise for coaches*	Example exercise for players**
Self-awareness	The aim is to support youth players to become aware of and reflect about their psychological and physical state.	Self-awareness is a basic step toward handling arousal and energy level. Being able to become conscious of one’s physical and psychological state enables the athlete to respond in an optimal way to the present situation.	Check-In, check-out	Note thoughts, emotions, and behaviors
Relaxation	The aim is to introduce different techniques for relaxation and relief in muscle tension	Conforming to the stress-injury model[35], injury risk depends on the stress response of the individual. A strong stress response has been shown to be related with injury risk[36]. The stress response can include muscular tension and attentional deficits. In order to buffer the stress response, it is proposed to actively work with relaxation and attentional techniques.	Progressive muscle relaxation	Progressive muscle relaxation, breathing exercises, music
Being in the present moment	The aim is increased awareness of one’s attention. This includes for example becoming aware when one’s attention wandered from the task at hand and being able to re-focus on it.		Being here and now, focus loop, mindful stretching	Being here and now, focus loop, mindfulness-based exercises
Prevent and handle stress	The aim is increased applied knowledge about strategies related to recovery, prevention of stress and coping with stressful situations.	Based on the stress-injury model[35], life stress is a major contributor to the stress response. High levels of negative life-event stress have been shown to be related with injury risk[36].	Recovery in practice, sleep	Recovery in practice, plan your recovery, sleep, my stress signals, problem solving, mindfulness exercise coming back to the present moment when stressed, weekly planning, screen time

*Note*: The number of different variations of the exercise/area is given in parenthesis

* In total 11 exercises; ** in total 25 exercises

The expert agreed on the distinction of exercises into the four principles and believed that the players’ exercises were meaningful and relevant. The expert’s feedback led to the following revisions: (1) To increase self-determination, instructions were adapted to encourage players to freely explore the exercises and pick exercises that are considered important for them, instead of recommending pre-defined training plans; (2) Some major (e.g., change exercise order, removal of one exercise) and minor changes (e.g., adapted wording and instructions). At the end of this process, the players’ module consisted of 25 exercises.

Overall, the expert found the exercises for coaches to be relevant and meaningful and believed these exercises had the potential to add a new aspect in the education of youth handball players in Sweden. The expert raised two points that were adapted accordingly: First, a new text was added to the coaches’ module with advice on how to communicate with players who answer with “red” (i.e., my physical and/or mental state is not good) to avoid reinforcing unhelpful behaviors. Second, a short section was added in the coach module to inform about the players’ exercises aimed to improve being in the present moment, and to encourage coaches to support players in executing these exercises at home. All experts agreed on the proposed psychological principles and on players’ exercises and exercises for coaches to do with their team(s). Two experts gave more detailed feedback that led to two minor changes.

Feedback from end-users (players) suggested they found the exercises meaningful and usable. The players stressed the importance to link exercises to performance enhancement, and not only injury prevention, to increase motivation. This led to a change in the instruction of four exercises. The exercise about the use of screens and its relationship with perceived stress was appreciated, and the players believed that this exercise was also important for younger players. Therefore, this exercise was also made available for the younger players. Also, based on the players’ feedback, some minor changes in wording were made to increase understanding of exercises.

### Mobile application

The intervention, i.e., information and training, was made available for each end-user group in the mobile application (Table 4). All modules included basic information and end-user-target additional information. The coach module included injury prevention physical and sport psychology exercises to be performed with their team(s). The physical exercises, and some of the sport psychology exercises, were integrated within warm-up and handball skills training. The player module included injury prevention handball-specific strength exercises, sport psychology exercises, and 12 handball-specific injury prevention exercises from the coach module (to perform, e.g., during holiday breaks). Consistent with requests conveyed by coaches and players, different programs were provided in the app over the season to support self-management and adoption. To increase motivation, players and coaches could change, add, progress the difficulty of exercises, and build their own program. Example programs for coaches to do with their team(s) and for players are given in Appendices D–G).

**Table 4:** End-user targeted I-PROTECT intervention, i.e., information and injury prevention training, made available in a mobile application (I-PROTECT GO), specifically developed for the I-PROTECT project, with modules for players, coaches, club administrators, and caregivers

**Basic information in all modules**			
• About I-PROTECT and I-PROTECT GO • Definition sports medicine and sport psychology related to current context • Current knowledge about injuries and benefits of injury prevention training • Physical principles of injury prevention including videos –Landing technique – Shoulder strength and throwing technique – Posture and core strength • Psychological principles of injury prevention – Self-awareness – Being in the present moment – Prevent and handle stress – Relaxation			
**Modules in I-PROTECT GO**			
**Players**	**Coaches**	**Club administrators**	**Caregivers**
**Additional information including specific advice**			
• Load management – Physical and mental load and recovery – Sleep – Food and fluids	• Motivation: autonomy, perceived competence, and relatedness (i.e., Self-Determination Theory) • Communication and feedback • Dialogue • Load management – Physical and mental load and recovery – Sleep – Food and fluids	• Motivation: autonomy, perceived competence, and relatedness (i.e., Self-Determination Theory) • Communication and feedback • Dialogue • Load management – Physical and mental load and recovery – Sleep – Food and fluids • Implementation including key components of strategies, processes, and actions (e.g., describe goals, identify possible barriers and solutions to address these, describe follow-up, maintenance, activities, roles and responsibilities)	• Motivation: autonomy, perceived competence, and relatedness (i.e., Self-Determination Theory) • Communication and feedback • Dialogue • Load management – Physical and mental load and recovery – Sleep – Food and fluids
**Training**			
**Age 13–14 years** • Strength and plyometric exercises (body weight or low weights/resistance) – 1 program including 8 exercises for legs, core, shoulders with possibility to change and/or add exercises • Sport psychology exercises – 1 program with 1–2 monthly exercises over 6 months • Handball-specific exercises – 8 exercises (legs n = 3, shoulder n = 4, core n = 1) **Age 15–17 years** • Strength and plyometric exercises (with weights) – 3 programs including 8 exercises for legs, core, shoulders with possibility to change and/or add exercises • Sport psychology exercises – 2 programs to choose from with 1–2 monthly exercises or 3–6 monthly exercises over 6 months • Handball-specific exercises including physical principles of injury prevention (from coach module) – 8 exercises (legs n = 3, shoulder n = 4, core n = 1)	**Age-related, i.e., 13–14 years and 15–17 years** • Handball-specific exercises and sport psychology exercises – 6 training periods (one period is used over approx. 6 weeks), each including 3 example programs for variation, thus, in total 18 example programs over a season. Each program includes 5–7 physical exercises for legs, shoulders, core, whereof 1–2 programs also include 1–2 sport psychology exercises. – 1–2 alternative exercises provided for each exercise so that coaches have the opportunity to change and/or progress difficulty of exercises – Possibility to build own program	• 1 program of handball-specific exercises from coach module • Example of strength exercises (13–14 years and 15–17 years) from player module • Example of sport psychology exercises from player module	• 1 program of handball-specific exercises from coach module • Example of strength exercises (13–14 years and 15–17 years) from player module • Example of sport psychology exercises from player module

### Feedback from early end-users

The overarching categories and coaches’ evaluation of the intervention in terms of facilitators and barriers are provided in Appendix H. Statements related to different aspects of the exercises in the mobile application are categorized into the theme *exercises*. The theme *set-up* refers to coaches’ statements regarding how the intervention was constructed. *Delivery* refers to how the content of the intervention was presented in the mobile application. Finally, the theme *implementation* includes statements about facilitators and barriers in the use of the intervention.

Overall, the players liked the mobile application, revealed by several positive comments during the interviews. The overarching categories that emerged from the analysis of the players’ interviews are shown in Appendix I. The theme *application* refers to factors related to how the application is built and presented. The theme *exercises* refers to the main content of the application, i.e., the handball-specific strength exercises and the sport psychology exercises. The theme *implementation* refers to factors related to the continued use of the application. In all categories, players’ statements revealed several facilitators and a few barriers.

Feedback from the club administrator, in terms of barriers, facilitators, and ideas for future improvement, is provided in Appendix J. Identified barriers were mostly about implementation (e.g., a challenge to start something new, unclear roles and responsibilities), while there were few for content and delivery (i.e., some exercises difficult, lack of knowledge of functions in app). Several facilitators were identified for content (e.g., comprehensive and sound content, proactive tool, and relevant exercises), delivery (app helpful and useful), and implementation (high priority, sustainable handball).

## Discussion

To our knowledge, this is the first study with an intervention that includes both physical and psychological aspects of injury prevention and that targets the individual, team, and organizational levels. A full version of the I-PROTECT intervention (information and training) was developed in a co-creating process involving researchers/experts (sports medicine, sport psychology, handball, physical therapy, and/or strength and conditioning), and end-users (coaches and players). Initial feedback from end-users identified several facilitators (e.g., relevant exercises, comprehensive content, helpful delivery) and some barriers (e.g., implementation).

Based on end-users’ needs identified in our first I-PROTECT project study [17], and building on the first pilot version [10], we incorporated injury prevention principles for upper and lower extremities into handball-specific exercises for regular handball practice. Available programs for youth handball players target either lower or upper limbs, and although effective under controlled conditions [7], each program takes 10–15 min to perform. Given that the main barrier to implementation is perceived time burden [19], it is not likely that multiple injury prevention programs will be implemented in the real-world setting. As end-users requested [10, 18], we included fewer exercises (n = 5–7) than in our pilot program (n = 7–10) and more variations in exercises. Also, exercises remained integrated within warm-up and skills training. Flexibility in mode of delivery is supported by a previous study reporting maintained effectiveness and increased compliance for the 11 + injury prevention program for soccer players if some exercises were performed during the beginning and some at the end of the training compared with performed at the beginning only [30].

We developed strength training informed by knowledge that such training is effective in improving muscle strength and functional performance in youth athletes [31], and that muscle strength enhances efficiency of movement and athletic performance and may reduce the risk of injury [32, 33]. Also, results from our previous studies including consensus reached among experts and end-users’ requests [10, 17] informed the training. The handball-specific strength training for youth players included multi-joint muscle strength and power exercises for lower extremity, upper extremity and core, and explosive strength to be performed by players at the gym (alone or with teammates). These components are in line with that recommended, with the aim to induce transfer effects on the specific handball-related skill performances [32]. General principles for programming and periodization were followed, as systematic variation of volume and intensity is most effective for long-term progression [34]. Each program would take 45–60 min to complete, and 1–3 training sessions per week were recommended. Given that training to failure may not be necessary to improve maximum muscular strength [34], we included instructions that players should be able to perform an additional 1–2 repetitions with good technique after each set. In line with that recommended [32, 34], younger players (13–14 years), were provided with multi-joint bodyweight exercises, whereof some with low loads (e.g., resistance bands).

Psychological injury prevention interventions are effective [28], yet still rare. Sport psychology exercises were developed based on the principles self-awareness, being in the present moment, relaxation, and prevent and handle stress. Stress and the individual’s response to stress are related to injury risk [35, 36]. It is thought that the stress response may lead to physiological (i.e., increased muscle tension) and attentional (i.e., disruptions in attention) changes, and interventions that buffer this stress response through physiological and attentional pathways are, therefore, recommended [35]. Exercises including the principles relaxation and being here and now, target these processes. As recommended [36], stress management training was included, as preventing and handling stress is a prerequisite to reduce the stress response. Finally, self-awareness forms the most central principle, as self-awareness is the underlying basis of, and a crucial precondition for, any sport psychology intervention [37]. As emerged in the workshop with the end-users, and in earlier research [19], motivation increases if injury prevention exercises also enhance performance. The four principles applied in our study are included in the mental performance competencies suggested in a recent framework [38]. This link to performance enhancement was therefore explicitly mentioned in the exercise description to increase participants’ motivation.

In line with previous research [19], facilitators included the broad selection and variation of exercises, the possibility to choose exercises, research-based exercises, and that the mobile application was easy to use. The coaches and the club administrator appreciated the sport psychology exercises, an aspect of injury prevention that is generally not used in Swedish handball. Time burden is one typical barrier for injury prevention training [19]. Players found it difficult to find time to do the strength and sport psychology exercises, and although the exercises that coaches do with their teams are integrated into regular practice, coaches still believed it took too much time from practice. Coaches also mentioned that time was needed to learn the exercises. To facilitate implementation of injury prevention training, educational activities [19] and support from the club [17] is needed.

The main strengths of this study include that: (i) identified barriers and facilitators from our previous studies informed the intervention, (ii) a holistic intervention was co-created with researchers/experts and end-users, (iii) behavioral and motivational theories were applied in the development of the intervention, and (iv) the intervention targeted end-users at different levels. There are some limitations to acknowledge. First, while the handball-specific exercises and some sport psychology exercises were integrated within regular handball training, as requested by end-users, the strength exercises and sport psychology exercises for players were to be performed outside handball practice, which places higher demands on the players’ own motivation to perform these exercises. Second, end-users’ involvement depended on the starting point for the different components of the intervention. Our previous studies, where end-users were involved [10, 17, 18], informed the development of the information and the extension of handball-specific exercises, so additional end-users were not included. The need to develop strength training and sport psychology exercises were identified in a previous study [10], and was completed in a series of workshops. Given that players and coaches were well familiar with strength training, researchers/experts and end-users development of this training together. In contrast, experts initiated the development of the sport psychology exercises, and end-users tested and evaluated them, because players and coaches were generally unfamiliar with this type of exercises. Third, although feedback from end-users was generally positive, only a small number of individuals were interviewed, and, thus, this data can only be considered preliminary. A larger number of end-users is needed to fully evaluate the intervention and delivery. Although our intervention was developed in co-design with end-users, this does not guarantee uptake and sustained use. Tailored implementation strategies, e.g., activities, that target the different stakeholders are needed to enhance the adoption, implementation, and sustainability of an intervention [29]. This is a subject for further study.

## Conclusions

A process that engaged end-users and researchers/experts to develop injury prevention training specifically for youth handball, generated information and training targeting end-users at the individual, team, and organizational levels. The intervention content (information and training) was made available in a mobile application, specifically developed for the project, with modules for players (information, strength and sport psychology exercises), coaches (information, physical and sport psychology exercises to integrate within handball practice), club administrators (information), and caregivers (information). Further studies will test and evaluate the intervention content and the mobile application.

### Electronic supplementary material

Below is the link to the electronic supplementary material.

Supplementary Material 1

## Data Availability

The data used in this study contains sensitive information about the study participants and they did not provide consent for public data sharing. The current approvals by the Swedish Ethical Review Authority (Reference numbers: 2014/713, 2020–02952, 2022-06148-02) does not include data sharing. A minimal data set could be shared by request from a qualified academic investigator for the sole purpose of replicating the present study, provided the data transfer is in agreement with EU legislation on the general data protection regulation and approval by the Swedish Ethical Review Authority. Contact information: Department of Health Sciences, Faculty of Medicine, Lund University Box 117, 221 00 Lund, Sweden. Contact address: DHSdataaccess@med.lu.sePrincipal investigator: Eva Ageberg, Department of Health Sciences, Faculty of Medicine, Lund University, PO Box 117, SE-221 00 Lund, Sweden. Email: eva.ageberg@med.lu.seSwedish Ethical Review Authority, Box 2110, 75 002 Uppsala, Sweden. Phone: +46 10 475 08 00.
