# Supplementary material for: Co-creating holistic injury prevention training for youth handball: Development of an intervention targeting end-users at the individual, team, and organizational levels
Source: BMC Sports Sci Med Rehabil. 2024 Jan 8;16:10. doi: 10.1186/s13102-023-00800-6 (PMC10773026; doi:10.1186/s13102-023-00800-6)
Supplement: Supplementary file 1 — Supplementary Material 1 [file 13102_2023_800_MOESM1_ESM.docx]

# Appendices

**Appendix A**. Barriers and facilitators regarding content and delivery of the intervention identified in previous studies (Ageberg et al 2019, 2022, Moesch et al 2022), categorized according to relevant topics of the Innovation domain of the updated Consolidated Framework for Implementation Research (CFIR) (Damschroder et al 2022). Because the first I-PROTECT project study (Ageberg et al 2019) identified facilitators, and not barriers, to support implementation of injury prevention training, focus is on facilitators.

| **CFIR Domain, topic and description** | **Barriers** | **Facilitators** |
| --- | --- | --- |
| **I. Innovation domain** |  |  |
| D. Innovation adaptability “*The degree to which the innovation can be modified, tailored, or refined to fit local context or needs”* | - Limited space and material - Be given a number of exercises without providing principles of injury prevention | - Knowledge on why (importance, benefits, and principles of injury prevention training) what (example of exercises) and how (clear instructions) - Information and training targeting the different end-users (players, coaches, club administrators, caregivers) - Emphasize that injury prevention training also enhances performance - Raise importance of recovery (rest and nutrition) - Age-related exercises - Informed coach communication - Create a positive and accepting training environment - Support from the club - Role models using/talking about the program |
| G. Innovation design  *"The degree to which the innovation is well designed and packaged, including how it is assembled, bundled, and presented"* | - Uninformed re. correct and incorrect movement technique - Uninformed on how to provide feedback - Some exercises too difficult - Lack of progression of difficulty - Too many new exercises at the same time - Having to choose from a bank of exercises only - Too repetitive and too time-consuming | - Exercises in pilot version generally practicable, relevant, fun, meaningful (handball-specific), new, and varying - Clear instructions re. why (purpose and relevance to handball, injury prevention, and performance) and how (written instructions and video of physical exercises) - Pairwise and group exercises fun - Knowledge on correct movement technique - Feedback to perform exercises correctly - Programs with selected exercises - Option to change/choose exercise and level of progression - Exercises for players to do during, e.g., holiday breaks - Role models, i.e., elite/professional and youth players demonstrate exercises on video - Exercises available in digital platform for all end-users - Perceived improvement over time |

**Appendix B**. Results from the three workshops in which handball-specific strength exercises were developed.

| **Workshop 1: Experts (strength and conditioning coaches, n=2; physical therapists, n=1) and end-users (coaches, n=2; players, n=6)** | **Workshop 2: Experts (strength and conditioning coaches, n=2; physical therapists, n=2)** | **Workshop 3: Experts (physical therapists, n=2)** |
| --- | --- | --- |
| Participants agreed on general principles and setup, type and example of exercises:  General principles   - Lower extremities: Hip-knee-foot alignment, and hip-width apart - Upper extremities: 1 front and 2 back exercises, and good posture - Mobility exercises in warm-up   Setup and type of exercises   - Closed chain - Tempo important, besides load - Work through whole movement - Super-sets (alternate 2 or 3 exercises, preferably contrasting exercises such as squats and jumps) - Jump exercises with focus on both jump and landing technique - Exercises in different planes (frontal, sagittal, transverse) - Double-leg and single-leg exercises - Use mirrors and peer-feedback   Examples of exercises   - Lower extremities: Nordic hamstrings, Hip-thruster, Romanian deadlift, Kettlebell swing, Squats with kettlebells, dumbbells, or trap-bar rather than barbell to facilitate correct technique, Step-up and step-down with dumbbells or barbell, Slide-exercises, Copenhagen adductor, Squat jumps, skate jumps, box jumps - Upper extremities and core: Bench press, Shoulder press, Overhead press, Facepull, Pulldown, Chins, Russian twist, Joystick with rotation, Dumbbell side bend, Medicine ball throw | Participants agreed on:   - Provide players with 3 different programs to choose from per week - Each program should include 8 exercises for whole body (lower extremity, upper extremity, core) - Provide each exercise with 2 alternative exercises (i.e., option to change exercise) - Each program should take 45–60 minutes - Recommend 1–3 training sessions per week - Sets: 3 - Repetitions: 10–12, 8–10, or 6–8. Change after 3 weeks - Load: should be able to perform an additional 1–2 repetitions with good technique after each set - Tempo (certain exercises): first part 3 seconds, 1–second paus, last part as quickly as possible, 1–second paus - Young players should be accompanied by an adult in the gym | Participants agreed on additional principles and type of exercises:   - Emphasize good posture in general - Add exercises in the frontal plane and with progression of difficulty - Hip abductors important for lower extremities - Include additional exercises for core and back - Exercises for players 13–14 years with body weight or low weight/resistance and focus on technique |
| Feedback from players regarding exercises and delivery to enhance motivation that had not emerged from previous studies:   - Free weights rather than machines - Exercises targeting muscle groups and body parts rather than separate muscles - Include balance training in complex exercises - Exercises for different player positions (back, wing, line players) - Instructions regarding sets, repetitions, pace, load |  |  |

**Appendix C**. Identified literature on sport psychology exercises for injury prevention.

**Intervention studies**

1. Edvardsson A, Ivarsson A, Johnson U. Is a cognitive-behavioural biofeedback intervention useful to reduce injury risk in junior football players? J Sports Sci Med. 2012 Jun;11(2):331-8.
2. Ivarsson A, Johnson U, Andersen MB, Fallby J, Altemyr M. It pays to pay attention: A mindfulness-based program for injury prevention with soccer players. J Appl Sport Psychol. 2015 Apr;27(3):319–34.
3. Johnson U, Ekengren J, Andersen MB. Injury prevention in Sweden: Helping soccer players at risk. J Sport Exerc Psychol. 2005 Mar;27(1):32–8.
4. Kerr G, Goss J. The effects of a stress management program on injuries and stress levels. J Appl Sport Psychol. 1996 Mar;8(1):109–17.
5. Kolt GS, Hume PA, Smith P, Williams MM. Effects of a stress-management program on injury and stress of competitive gymnasts. Percept Mot Skills. 2004 Aug;99(1):195–207.
6. Olmedilla-Zafra A, Rubio VJ, Ortega E, García-Mas A. Effectiveness of a stress management pilot program aimed at reducing the incidence of sports injuries in young football (soccer) players. Phys Ther Sport. 2017 Mar;24:53–9.
7. Perna FM, Antoni MH, Baum A, Gordon P, Schneiderman N. Cognitive behavioral stress management effects on injury and illness among competitive athletes: A Randomized Clinical trial. Ann Behav Med. 2003 Jan;25(1):66–73.
8. Tranaeus U, Johnson U, Engström B, Skillgate E, Werner S. A psychological injury prevention group intervention in Swedish floorball. Knee Surg Sports Traumatol Arthrosc. 2015 Nov;23(11):3414–20.
9. Tranaeus U, Johnson U, Ivarsson A, Engström B, Skillgate E, Werner S. Sports injury prevention in Swedish elite floorball players: evaluation of two consecutive floorball seasons. Knee Surg Sports Traumatol Arthrosc. 2015 Mar;23(3):899–905.

**Overview studies**

1. Gledhill A, Forsdyke D, Murray E. Psychological interventions used to reduce sports injuries: a systematic review of real-world effectiveness. Br J Sports Med. 2018 Aug;52(15):967.
2. Ivarsson A, Johnson U, Andersen MB, Tranaeus U, Stenling A, Lindwall M. Psychosocial factors and sport injuries: Meta-analyses for prediction and prevention. Sports Med. 2017 Feb;47(2):353–65.
3. Johnson U. Psychosocial antecedents of sport injury, prevention, and intervention: An overview of theoretical approaches and empirical findings. Int J Sport Exerc Psychol. 2011 Feb;5(4):352–69.
4. Johnson U, Tranaeus U, Ivarsson A. Current status and future challenges in psychological research of sport injury prediction and prevention: a methodological perspective. Revista de Psicología del Deporte. 2014 Jul; 23(2):401-09.
5. Petterson H, Olson BL. Effects of mindfulness-based interventions in high school and college athletes for reducing stress and injury, and improving quality of life. J Sport Rehabil. 2017 Nov;26(6):578–87.
6. Tranaeus U, Ivarsson A, Johnson U. Evaluation of the effects of psychological prevention interventions on sport injuries: A meta-analysis. Sci Sports. 2015 Dec;30(6):305–13.

**Book**

1. Biegel, GM. Stress reduction workbook for teens. Mindfulness skills to help you deal with stress. 2 ed. Oakland, Calif: New Harbinger Publications; 2017.

**Appendix D**. Example program 1 including handball-exercises (main focus legs, core, shoulder) and psychological exercises (self-awareness) for younger (age 13–14 years) and older (age 15–17 years) players for coaches to do at handball practice (warm-up and skills training) with their team(s), available in the coach module. A handball is included in most physical exercises.

| **Main focus** | **Exercise description (level of progression)** | **Alternative exercises (level of progression) players 13–14 years** | **Alternative exercises (level of progression) players 15–17 years** |
| --- | --- | --- | --- |
| Legs (W) | Single-leg balance with drop and catch ball (1) | Starting position lifted leg 90 degrees in hip and knee (2) | Starting position lifted leg 90 degrees in hip and knee (2) |
| Legs (W) | Run with foot plant (1) | Forward lunges – lift front leg 90 degrees hip and knee (1) | Forward lunges – lift front leg 90 degrees hip and knee (1); Forward lunges – lift front leg – toe-raise standing leg (2) |
| Core (W) | Single-leg balance with arm wrestling and ball, pairwise (1) | Plank on knees with arm wrestling and ball, pairwise (1) | Plank on knees with arm wrestling and ball, pairwise (1); Plank on feet with arm wrestling and ball, pairwise |
| Shoulder (W) | Standing external rotation with resistance band (1) | Standing external rotation with ball (1); pairwise bow and arrow with resistance band (1) | Standing external rotation with ball (1); pairwise bow and arrow with resistance band (1) |
| Legs (H) | Slow motion feint with elevated arm (1) | Pairwise slow motion feint with elevated arm and push (2) | Pairwise slow motion feint with elevated arm and push (2) |
| Legs (H) | Running – jumping – landing on both feet (1) | Jumping and cutting while dribbling ball – landing on both feet (2) | Jumping and cutting while dribbling ball – landing on both feet (2); High speed jumping and cutting while dribbling ball – landing on both feet (3) |
| Self-awareness | Check-in | NA | NA |
| Self-awareness | Check-out | NA | NA |

W=warm-up; H=handball skills training; NA=not applicable

**Appendix E**. Example program strength training for younger (age 13–14 years) and older (age 15–17 years) players in player module.

| **Players 13–14 years (body weight or low weight/resistance)** | | | **Players 15–17 years (weight training)** | | | |
| --- | --- | --- | --- | --- | --- | --- |
| **Main body part** | **Exercise description** | **Alternative exercises** | **Main body part** | **Exercise description** | **Super-set** | **Alternative exercises** |
| Legs | Squats on both legs | Single-leg squats; Forward lunge with upper body rotation | Legs | Squats with kettlebells/dumbbells | 1 | Squats with barbell; Deadlift with trap-bar |
| Legs | Backward lunges | Split-squats; split-squats with back leg on bench | Jump/explosive strength | Standing long jump(s) | 1 | Box-jumps (take-off from one leg or both legs, with or without rotation); Death jumps |
| Legs | Side step-down | Crab walking with resistance band | Leg | Romanian deadlift | 2 | Single-leg Romanian deadlift; Kettlebell swing |
| Jump/explosive strength | Jumping from a step board, landing on floor with both feet | Skate-jumps; Single-to double-leg jumps (take-off on one leg – rotate 90 degrees – land on both feet) | Upper body | Seated rowing | 2 | Standing rowing; Face pull |
| Upper body | Side lying external rotation with dumbbell | Wall shoulder press (back); external rotation with resistance band standing position | Legs | Side step-down | 3 | Side step-down with weight plate; Copenhagen adductor |
| Upper body | Wheelbarrow | Push-ups; side-to-side push-ups | Upper body | Standing single-arm shoulder press | 3 | Alternating single-arm shoulder press; Rotational shoulder press |
| Upper body | Sitting rowing with resistance band | Single-arm row with resistance band standing position; Pull-ups | Core | Dead bug | NA | 2 arm 2 leg dead bug; dumbbell side bend |
| Core | Dead bug | Seated rotation with medicine ball (pairwise); Side plank | Legs | Side lying hip abductors (“Jane Fonda”) | NA |  |

**Appendix F**. Example program sport psychology for younger (age 13-14 years) and older (age 15-17 years) players in player module.

| **Month** | **Exercise description** | **Principle** |
| --- | --- | --- |
| 1 | Being here and now | Being in the present moment |
|  | Progressive muscle relaxation | Relaxation |
| 2 | Focus loop | Being in the present moment |
|  | Deep breathing | Relaxation |
| 3 | Body scan | Being in the present moment |
| 4 | Recovery in practice | Prevent and handle stress |
|  | My sleep | Prevent and handle stress |
| 5 | Land in the present moment | Being in the present moment |
|  | Weekly planning | Prevent and handle stress |
| 6 | Note thoughts, emotions, and behaviors | Self-awareness |

Note: This program is the recommended example program for 13-14 years old players; while players age 15-17 years can choose between this program and another program with more exercises.

**Appendix G**. Screenshots from mobile application I-PROTECT GO (only available in Swedish). Tailored example program (A) and example of physical exercise (B) from coach module, and tailored example strength training program (C) and example of sport psychology exercise (D) from player module. To support self-management, adoption, and motivation, several programs are available, and end-users can change, add, and progress the difficulty of exercises.

**Appendix H**. Overarching categories and coaches’ (n=6) evaluation of facilitators and barriers for using the intervention and mobile application.

|  | **CATEGORY** |  |
| --- | --- | --- |
| **Facilitators** |  | **Barriers** |
| **Appreciated exercises**   - - Handball-specific - - Sport psychology   **Confidence in research-based exercises** | EXERCISES | **Difficult exercises** |
| **Freedom to choose and decide upon exercise/program appreciated**  **Sport psychology as an additional topic appreciated**  **Variation through new and many exercises appreciated** | SET-UP | **Suggested exercises in program took too long time** |
| **Appreciated and comprehensible application**  **“Knowing the why” in exercise description**  **Valuable education**  **Videos appreciated** | DELIVERY | **Missing knowledge about content and functions in application** |
| **Clear roles and responsibilities within the team**  **Ideas for future improvement**   - - Clear lead from the club - - Education on site and during regular coach education - - Set goals for and monitor implementation - - Suitable timing of intervention start and implementation during practice | IMPLEMENTATION | **Bad and tight timing at start of the intervention**  **Unclear roles and responsibility within the coach team**  **Missing resources**  **Time-consuming and challenging to change to something new**  **Unclear ways of communication** |

**Appendix I**. Overarching categories and players’ (n=3) evaluation of facilitators and barriers for using the intervention and mobile application.

|  | **CATEGORY** |  |
| --- | --- | --- |
| **Facilitators** |  | **Barriers** |
| Appreciate autonomy  Appreciate layout  Appreciate variation  Appreciate video  Ease of use of application | MOBILE  APPLICATION | Don’t understand purpose of exercise  Technical problems |
| Confidence in exercise  Feels good to do exercise  Preparatory strength exercises good to secure technique | EXERCISES | Sport psychology exercises could be more fun |
| Possible to perform at home – alone  Shared experience with teammates is appreciated  Feeling improvement | IMPLEMENTATION | Lack of time |

**Appendix J**. Feedback on the intervention (content, delivery, implementation) from the club administrator (n=1).

|  | **Barriers** | **Facilitators** | **Ideas for future improvement** |
| --- | --- | --- | --- |
| Content | - Some exercises difficult | - Comprehensive (e.g., handball-specific, sport psychology, nutrition, sleep) and sound (research-based) content - Proactive tool for coaches, easy to understand how to prevent injuries - Relevant exercises - Sport psychology as an additional topic - Checklist for implementation valuable |  |
| Delivery | - Lack of knowledge about some functions in app | - The app is useful, easy to use, and has helpful and clear instructions and videos - Valuable education - The app is an education for coaches - Fulfills what coaches need to implement injury prevention training - Option to choose exercises/programs - Variation of new and several exercises | - Add functions in app, e.g., ping a specific exercise, rate exercises - Improve app layout and graphics |
| Implementation | - Time-consuming - Challenging to change to something new - Other priorities - Dissemination from club - Unclear roles and responsibilities in club - Sustained use - Lack of resources - Unclear ways of communication - Timing of start | - Support from club at startup - Club takes responsibility to implement injury prevention training - Intervention fulfills what coaches need to implement injury prevention training - Integrated within handball practice - High priority, i.e., “low hanging fruit” - Sustainable handball - Timely issue - Clear roles and responsibilities within the team | - Information meetings - Educational films - Clear support from club - Appoint a club administrator who is responsible for implementation in the club and supports coaches - Supportive material for clubs at startup and for sustained use (checklists timelines and flow charts) - Recurring information within club - Annual plan - Clear priority from club - Select a suitable timing to start - Include in coach education |
